# A case report of successful implantation of a leadless pacemaker via the left internal jugular vein in a patient with mirror-image dextrocardia and absence of the inferior vena cava’s hepatic segment

**DOI:** 10.3389/fcvm.2025.1701484

**Published:** 2026-01-05

**Authors:** Yue Bao, Hongwei Yi, Hongwei Han

**Affiliations:** Department of Cardiology, Wuhan Asia Heart Hospital, Wuhan, China

**Keywords:** leadless pacemaker, mirror-image dextrocardia, hepatic segment interruption of theinferior vena cava, azygos continuation, internal jugular approach

## Abstract

Mirror-image dextrocardia combined with absence of the hepatic segment of the inferior vena cava (IVC) and continuation of the azygos vein represents an extremely rare dual anatomical variation that significantly increases the technical complexity of leadless pacemaker (LP) implantation. This paper presents a 28-year-old male patient who required permanent pacing therapy due to third-degree atrioventricular block. Preoperative computed tomography angiography (CTA) clearly identified the aforementioned malformations. A Micra AV leadless pacemaker was subsequently implanted successfully via the left internal jugular vein. During the procedure, image mirroring technology was employed to assist the operation, ensuring precise deployment and stable parameters. This case marks the world's first successful LP implantation via the internal jugular vein in a patient with such complex anatomical variations, offering a novel, safe, and feasible approach for similar patients.

## Introduction

Mirror-image dextrocardia is a rare congenital malformation, with an incidence of approximately 0.01%, is characterized by the heart's location in the right thoracic cavity and a mirror-image arrangement of the atria, ventricles, and great vessels relative to their normal anatomic positions.

It is frequently associated with other cardiovascular anomalies, which notably increases the difficulty of interventional procedures (1, 2). LPs have emerged as a preferred option for certain patients because they obviate the need for leads and pockets (3). However, when accompanied by the absence of the hepatic segment of the IVC and continuation of the azygos vein (incidence of around 0.6%), the conventional femoral vein approach often fails due to pronounced tortuosity and angulation (4, 5). Although some studies have explored alternative routes via the azygos vein or internal jugular vein (2, 4, 5), there have been no reports of LP implantation via the internal jugular vein in the context of mirror-image dextrocardia. In this case, preoperative CTA precisely identified the dual anatomical variations. The successful implantation of a Micra AV device via the left internal jugular vein, assisted by image mirroring technology during the procedure, resulted in optimal pacing parameters. This marks the world's first such case and provides a novel strategy for patients with complex anatomical conditions.

## Case report

A 28-year-old male, Eighteen years prior, he was diagnosed at an outside hospital with ventricular septal defect (VSD) combined with mirror-image dextrocardia and absence of the hepatic segment of the IVC.And he underwent surgical repair for VSD. Postoperatively, he developed third-degree atrioventricular block (AVB), which was not treated further. On admission, physical examination revealed no significant abnormalities, and all laboratory test results were within normal limits.

**Figure 1:**
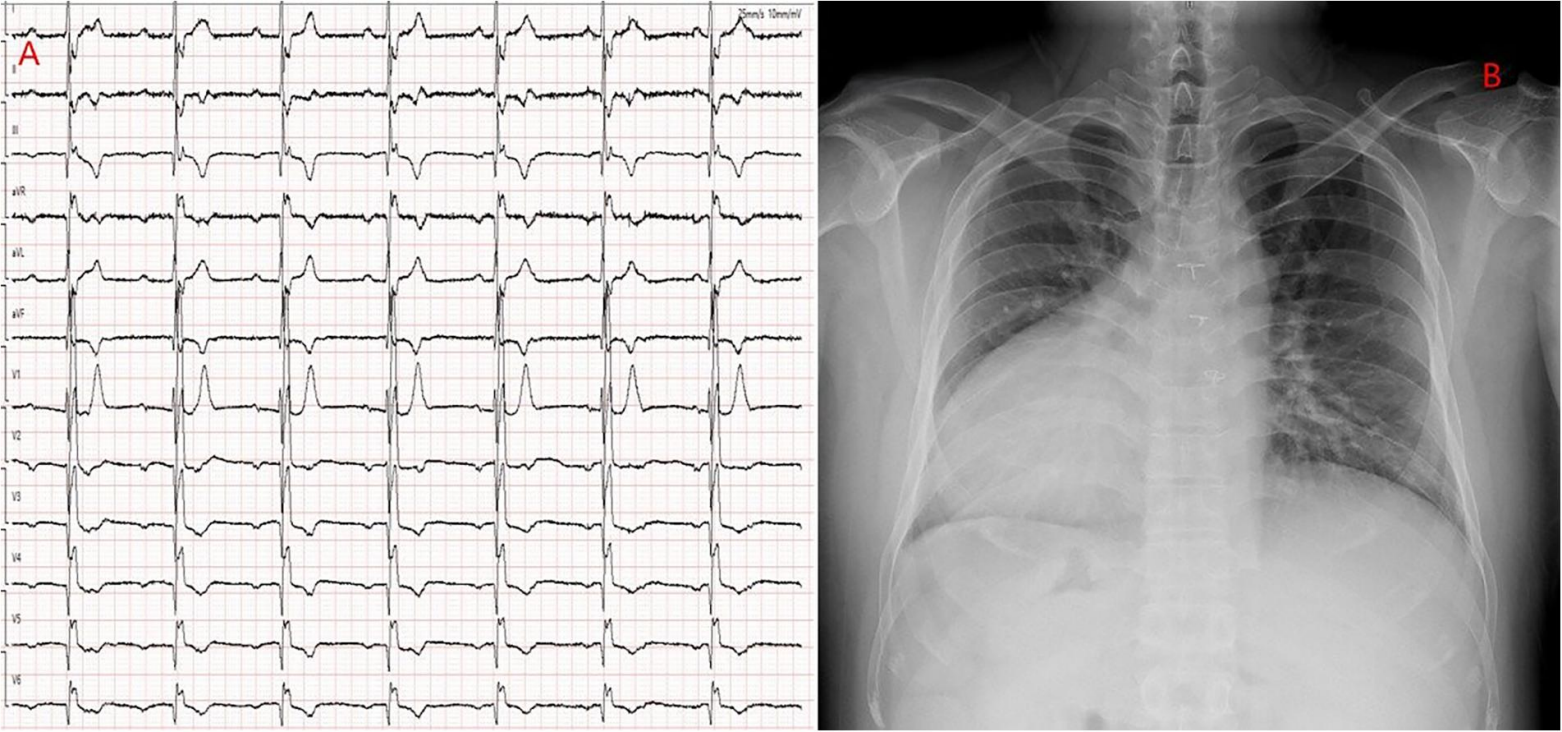
**(A)** ECG with reversed limb leads: Shows third-degree atrioventricular block and complete right bundle branch block. **(B)** Chest x-ray: Demonstrates a mirrored right-sided heart.

**Figure 2:**
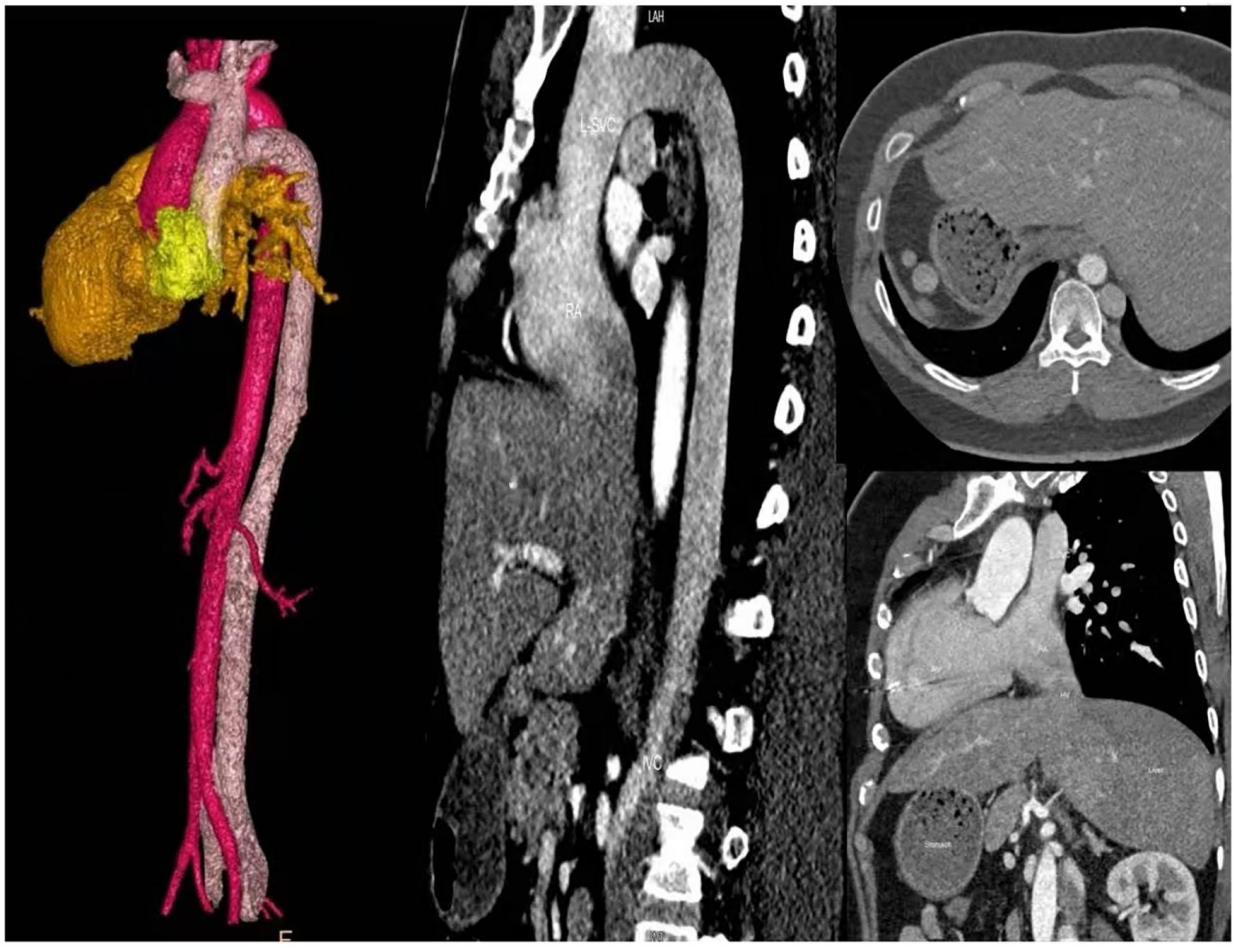
Chest and abdominal large vessel CTA: Absence of the hepatic segment of the inferior vena cava (IVC); the left-sided azygos vein is thickened and extends to the right superior vena cava (SVC); hepatic veins drain directly into the right atrium.

Echocardiography revealed mirror-image dextrocardia, a post-VSD repair status, and no evidence of ventricular-level shunting. Electrocardiography (performed with left and right hand electrodes reversed) demonstrated sinus rhythm, third-degree AVB, junctional escape rhythm, and complete right bundle branch block (Figure 1A). Chest radiography confirmed mirror-image dextrocardia (Figure 1B). CTA of the great vessels showed that the IVC was located on the left side of the aorta, with absence of its hepatic segment. The IVC ascended and connected to the right superior vena cava via a dilated azygos vein. The suprarenal portion of the IVC exhibited luminal stenosis, with the narrowest diameter measuring approximately 8.5 mm, while the hepatic veins drained directly into the right atrium (Figure 2). The patient fulfilled the Class I indication for pacemaker implantation (6). However, he declined a conventional lead-based pacing system and instead opted for leadless pacemaker implantation.

**Figure 3:**
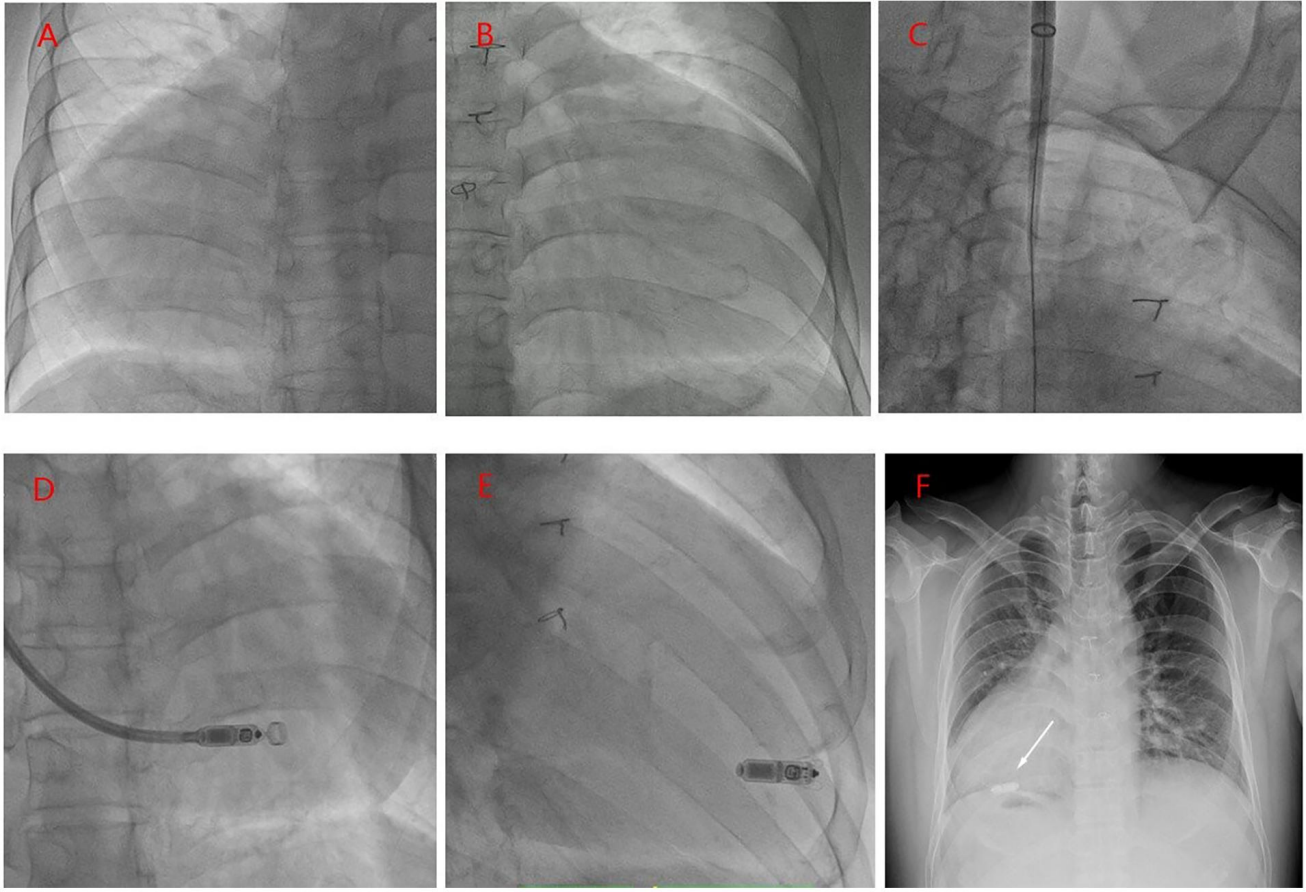
**(A)** RAO 30° fluoroscopy: Confirms a right-sided heart. **(B)** RAO 30° digital mirror view. **(C)** A 25 F delivery sheath was inserted via the left internal jugular vein, with its tip positioned in the right atrium. **(D)** A pre-loaded Micra AV controllable curved delivery system was advanced through the sheath, traversing the tricuspid valve and entering the anatomical right ventricle. **(E)** The leadless pacemaker was gradually deployed, with its distal tip anchored in the mid to lower ventricular septum. **(F)** Postoperative chest x-ray: The leadless pacemaker (white arrow) is correctly positioned against the mid-low ventricular septum without evidence of displacement.

A routine surgical procedure was carried out, including disinfection and draping. Fluoroscopy was utilized to verify the mirror-image positioning of the heart (Figure 3A). To align the operator's field of view with the conventional anatomical orientation, the x-ray image was horizontally mirrored using computer adjustments (Figure 3B).

A 6F sheath was introduced into the left femoral vein, and a temporary pacing electrode was placed. However, attempts to maneuver the electrode through the tortuous and angulated azygos vein to the superior vena cava (SVC) were unsuccessful. Subsequently, a 9F sheath was inserted into the right femoral vein, and angiography of the IVC and azygos vein was performed using a pigtail catheter. The imaging revealed that the azygos vein extended to the level of the tracheal bifurcation before turning and connecting to the right atrium. Despite repeated adjustments, positioning the temporary pacemaker into the right ventricle remained challenging. Given the long and angulated delivery path, it was anticipated that the MI2355A delivery sheath would not navigate safely through the route. As a result, the femoral approach was abandoned. Instead, the left internal jugular vein was punctured, and a guidewire was successfully advanced into the right atrium. After sequential dilation, the MI2355A delivery sheath was inserted (Figure 3C).

- The pre-loaded Micra AV leadless pacemaker (model MC1AVR1), equipped with a steerable delivery system, was introduced through the sheath and advanced through the tricuspid valve into the right ventricle. Angiography performed via the sheath confirmed that the pacemaker tip was securely positioned against the mid-lower portion of the ventricular septum (Figure 3D). The device was then gradually deployed (Figure 3E). Programmed parameters included a sensing amplitude of 12.5 mV, a pacing threshold of 0.5 V at 0.24 ms pulse duration, and an impedance of 750 Ω. Following wire traction and severance, reprogramming confirmed stable and consistent parameters. Subsequently, the delivery system and sheath were removed, the puncture site was sutured, and a compression dressing was applied, marking the completion of the procedure. Postoperative chest radiography revealed no signs of device displacement (Figure 3F), and Post-implant pacemaker programming demonstrated stable parameters. One month after implantation, the patient reported no symptoms.

## Discussion

The incidence of mirror-image dextrocardia is approximately 0.01%, and 23%–70% of cases are associated with other cardiovascular malformations (1). The absence of the hepatic segment of the IVC, with continuation via the azygos vein, occurs in about 0.6% of individuals. Although this anomaly is typically asymptomatic, it can lead to significant elongation and sharp angulation along the femoral vein access path, making it challenging for the leadless pacemaker delivery system to navigate (4, 5). For preoperative evaluation of complex congenital heart diseases, high-resolution CTA is widely recommended by clinical guidelines (2, 3).

Published literature suggests that the success rate of LP implantation in patients with IVC anomalies is closely related to the angle between the azygos vein and the SVC, as well as the support provided by the delivery sheath. When this angle exceeds 90°, the failure rate of the femoral venous approach can reach up to 57%, whereas the SVC approach achieves a success rate of nearly 100% (4, 5, 7–9). When the femoral approach is not feasible, the internal jugular vein serves as a viable alternative access route. Oliveira et al. (4) first described LP implantation via the azygos vein; however, this technique required a stiff guidewire, which increased procedural risk. Guo et al. (5) later demonstrated successful implantation through the right internal jugular vein, confirming its safety. In this case, we used computer imaging technology: through the “Patient Orientation”function, we displayed the images in a prone position.the patient underwent image mirroring to align catheter manipulation with the operator's field of vision, thereby improving procedural safety and reducing fluoroscopy time. Furthermore, the anatomical right ventricle (systemic ventricle) contains abundant trabeculae that provide favorable conditions for wing anchor fixation. However, care should be taken to avoid the moderator band to prevent an elevated pacing threshold (10). Performing intra-sheath angiography during the procedure allows for real-time assessment and timely detection of potential myocardial perforation.Limitations: The lack of long-term follow-up in this single case highlights the need for larger studies to validate fixation reliability and parameter stability.

This case is exceptionally rare. It represents the first successful implantation of a Micra AV pacemaker in a patient with a mirror-image right-sided heart and absence of the hepatic segment of the inferior vena cava, performed via the left internal jugular vein. During the procedure, the use of image mirroring technology reduced fluoroscopy time and ensured accurate device deployment, demonstrating a safe and viable alternative for patients with complex anatomical variations.

## Data Availability

The original contributions presented in the study are included in the article/Supplementary Material, further inquiries can be directed to the corresponding authors.
